# Pre-transplant Type 2 Diabetes Mellitus Is Associated With Higher Graft Failure and Increased 5-Year Mortality After Heart Transplantation

**DOI:** 10.3389/fcvm.2022.890359

**Published:** 2022-06-09

**Authors:** Rasmus Rivinius, Carolin Gralla, Matthias Helmschrott, Fabrice F. Darche, Philipp Ehlermann, Tom Bruckner, Wiebke Sommer, Gregor Warnecke, Stefan Kopf, Julia Szendroedi, Norbert Frey, Lars P. Kihm

**Affiliations:** ^1^Department of Cardiology, Angiology and Pneumology, Heidelberg University Hospital, Heidelberg, Germany; ^2^Partner Site Heidelberg/Mannheim, German Center for Cardiovascular Research, Heidelberg, Germany; ^3^Institute of Medical Biometry, University of Heidelberg, Heidelberg, Germany; ^4^Department of Cardiac Surgery, Heidelberg University Hospital, Heidelberg, Germany; ^5^Department of Internal Medicine I and Clinical Chemistry, Heidelberg University Hospital, Heidelberg, Germany; ^6^German Center for Diabetes Research, Neuherberg, Germany

**Keywords:** diabetes mellitus, graft failure, HbA1c, heart transplantation, mortality, survival

## Abstract

**Aims:**

Cardiac transplant recipients often suffer from type 2 diabetes mellitus (T2DM) but its influence on graft failure and post-transplant mortality remains unknown. The aim of this study was to investigate the long-term effects of pre-transplant T2DM in patients after heart transplantation (HTX).

**Methods:**

This study included a total of 376 adult patients who received HTX at Heidelberg Heart Center between 01/01/2000 and 01/10/2016. HTX recipients were stratified by diagnosis of T2DM at the time of HTX. Patients with T2DM were further subdivided by hemoglobin A1c (HbA1c ≥ 7.0%). Analysis included donor and recipient data, immunosuppressive drugs, concomitant medications, post-transplant mortality, and causes of death. Five-year post-transplant mortality was further assessed by multivariate analysis (Cox regression) and Kaplan–Meier estimator.

**Results:**

About one-third of all HTX recipients had T2DM (121 of 376 [32.2%]). Patients with T2DM showed an increased 5-year post-transplant mortality (41.3% versus 29.8%; *P* = 0.027) and had a higher percentage of death due to graft failure (14.9% versus 7.8%; *P* = 0.035). Multivariate analysis showed T2DM (HR: 1.563; 95% CI: 1.053–2.319; *P* = 0.027) as an independent risk factor for 5-year mortality after HTX. Kaplan–Meier analysis showed a significantly better 5-year post-transplant survival of patients with T2DM and a HbA1c < 7.0% than patients with T2DM and a HbA1c ≥ 7.0% (68.7% versus 46.3%; *P* = 0.008) emphasizing the clinical relevance of a well-controlled T2DM in HTX recipients.

**Conclusion:**

Pre-transplant T2DM is associated with higher graft failure and increased 5-year mortality after HTX.

## Introduction

Type 2 diabetes mellitus (T2DM) is a common comorbidity in patients with advanced heart failure and is often associated with a variety of extracardiac diseases such as obesity, impaired wound healing with increased risk of infection, thromboembolic complications, and renal dysfunction (1–8). Given these risk factors, T2DM is considered a relative contraindication for listing for heart transplantation (HTX), depending on the patient’s diabetes status and severity of end-organ damage (4–8).

Encouraged by reasonable post-transplant outcomes of patients without evidence of end-organ damage from T2DM at the time of HTX (9–12), an increasing number of patients with T2DM were listed for HTX and subsequently transplanted. This development was supported by the growing number of patients with advanced heart failure and T2DM over the last decades (4–8). These early studies, however, included rather small numbers of carefully selected diabetic patients not necessarily reflecting clinical reality (9–12). It is therefore not surprising that recent literature is inconclusive as some studies found an elevated post-transplant mortality in patients with pre-transplant T2DM (13–16), whereas others could not observe such effect (17–21). Differences in study design, sample size, length of follow-up and analyzed post-transplant outcomes may have contributed to these inconsistencies (13–21). In addition, it should be noted that there was a distinct change in the composition of HTX study populations over time, since the reported rates of diabetic HTX recipients increased markedly from 13.7% to 18.3% in former studies (22, 23) up to 28.8–30.7% in recent studies (24, 25).

Another important aspect is the clinical management of patients with T2DM as a poorly controlled hemoglobin A1c (HbA1c) may be associated with increased post-transplant mortality. Furthermore, there might be an essential difference between T2DM patients with oral anti-diabetic medications and T2DM patients with insulin therapy. Yet, these questions have not been sufficiently answered in the literature. We therefore sought to investigate the effects of pre-transplant T2DM on survival and causes of death after HTX in a large contemporary population of HTX recipients.

## Patients and Methods

### Patients

We performed this study in accordance with the ethical standards of the Declaration of Helsinki in its current form. Approval was granted by the institutional review board (IRB) of Heidelberg University (ethical approval number: S-286/2015, Version 1.2, 28-07-2020). Written informed consent was obtained from patients for inclusion in the Heidelberg HTX Registry allowing the clinical and scientific use of data. According to the ethical approval, no additional written informed consent was required for this observational study as merely routine clinical data were analyzed (26–35).

We screened all adult patients (≥18 years) for pre-transplant T2DM who received HTX at Heidelberg Heart Center, Heidelberg, Germany, between 01/01/2000 and 01/10/2016. Patients with type 1 diabetes mellitus or other forms of diabetes than T2DM were excluded. We also did not include patients who received a second HTX. The remaining patients were stratified by diagnosis of T2DM at the time of HTX: patients with T2DM at the time of HTX (“T2DM group”) and patients without T2DM at the time of HTX (“no T2DM group”). Patients with T2DM at the time of HTX were further divided into patients with and without insulin therapy as well as into patients with a HbA1c < 7.0% at the time of HTX and patients with a HbA1c ≥ 7.0% at the time of HTX.

### Follow-Up

Post-transplant follow-up was performed according to Heidelberg Heart Center’s routine clinical protocol. After hospital discharge, patients were monthly seen at the HTX outpatient-clinic during the first six post-transplant months, then bimonthly until the end of the first year after HTX, and thereafter three to four times annually (with additional visits when clinically required). Routine follow-up included medical history, physical examination, 12-lead electrocardiogram, echocardiography, endomyocardial biopsy, and blood tests including immunosuppressive drug monitoring (26–35).

### Post-transplant Medications

Post-transplant medication including immunosuppressive drug therapy was administered according to Heidelberg Heart Center’s usual standard of care. Patients perioperatively received an anti-thymocyte globulin-based immunosuppression induction therapy. Cyclosporine A and azathioprine were administered as the initial immunosuppressive regimen prior to 2001. From 2001 onward, mycophenolate mofetil subsequently replaced azathioprine, and tacrolimus consecutively replaced cyclosporine A from 2006 onward. Steroids were tapered incrementally during the initial post-transplant months and were discontinued 6 months after HTX (unless clinically needed) (26–35).

### Statistical Analysis

Data were analyzed with SAS (Version 9.4, SAS Institute, Cary, NC, United States) and expressed as count (n) with percentage (%) or as mean ± standard deviation (SD). For measures of association, difference of mean or hazard ratio (HR) with 95% confidence interval (CI) were used. Depending on the variable type (categorical variables or continuous variables) and the underlying question, we applied chi-squared test, Fisher’s exact test, Student’s *t*-test, Mann–Whitney *U*-test, analysis of variance (ANOVA), or Kruskal–Wallis test, as appropriate. The Kaplan–Meier estimator was used to graphically show 5-year post-transplant survival. A *P*-value of < 0.050 was considered statistically significant (26–35).

The primary outcome of this study was 5-year mortality after HTX which was compared between patients with and without T2DM at the time of HTX. We could obtain 5-year follow-up data from all patients requiring no censoring. Five-year post-transplant mortality of patients with T2DM was further assessed by stratification into patients with and without insulin therapy as well as into patients with a HbA1c < 7.0% at HTX and patients with a HbA1c ≥ 7.0% at HTX. Causes of death within 5 years after HTX were grouped into the following categories: graft failure, acute rejection, infection/sepsis, malignancy, and thromboembolic event/bleeding. We applied univariate analyses to search for intergroup differences including recipient data, previous open-heart surgery, principal diagnosis for HTX, donor data, transplant sex mismatch, perioperative data, immunosuppressive drug therapy, and concomitant medications. Analysis of 5-year mortality after HTX further included a multivariate analysis (Cox regression model) to investigate the impact of the eight variables which were statistically significant in the univariate analysis: recipient age (years), recipient body mass index (kg/m^2^), recipient arterial hypertension (in total), recipient dyslipidemia (in total), recipient T2DM (in total), recipient previous coronary artery bypass graft (CABG) surgery (in total), ischemic cardiomyopathy (CMP) as principal diagnosis for HTX (in total), and cardiac amyloidosis as principal diagnosis for HTX (in total). We did not include additional parameters in this multivariate analysis for 5-year mortality after HTX in order to avoid biased regression coefficients and to ensure a stable number of events (deceased patients) per analyzed variable. Given the long study period (01/01/2000–01/10/2016), we additionally performed a sensitivity analysis to test the robustness of our findings and to examine a possible era effect using a subgroup of patients with tacrolimus and mycophenolate mofetil as the immunosuppressive drug regimen was changed from 2006 onward (26–35).

## Results

### Baseline Characteristics of Patients With Type 2 Diabetes Mellitus at Heart Transplantation

This study included a total of 376 HTX recipients. About one-third of these patients (121 of 376 [32.2%]) had T2DM at the time of HTX. Patients with T2DM at the time of HTX were further divided into patients with a HbA1c < 7.0% at the time of HTX (67 of 121 [55.4%]) and patients with a HbA1c ≥ 7.0% at the time of HTX (54 of 121 [44.6%]).

Comparison of recipient data showed a higher age (*P* < 0.001), a higher body mass index (*P* < 0.001), a higher percentage of arterial hypertension (*P* < 0.001), and a higher percentage of dyslipidemia (*P* < 0.001) in the T2DM group. We did not observe a statistically significant difference between both groups concerning recipient male sex, chronic obstructive pulmonary disease, or severe chronic kidney disease (all *P* ≥ 0.050). Further evaluation of end-organ damage and clinical status of patients at the time of HTX showed no statistically significant difference between patients with or without T2DM concerning total bilirubin, hemoglobin, hospitalization before HTX, days on waiting list, high urgency status on waiting list, inotropic support, intra-aortic balloon pump, or initial hospital stay after HTX (all *P* ≥ 0.050).

In terms of principal diagnoses for HTX, significantly more patients with ischemic CMP were found in the T2DM group (*P* < 0.001), whereas significantly more patients with cardiac amyloidosis (*P* < 0.001) were observed in the opposite group. In addition, patients with T2DM had a significantly higher percentage of CABG surgery before HTX (*P* = 0.003). Baseline characteristics stratified by T2DM at HTX are shown in Table 1.

**TABLE 1:** Baseline characteristics – stratified by T2DM at HTX.

Parameter	All (*n* = 376)	T2DM (*n* = 121)	No T2DM (*n* = 255)	Difference	95% CI	*P*-value
**Recipient data**						
Age (years), mean ± SD	51.9 ± 10.4	56.0 ± 7.3	49.9 ± 11.0	6.1	4.2–8.0	<0.001*
Male sex, *n* (%)	287 (76.3%)	99 (81.8%)	188 (73.7%)	8.1%	–0.6 – 16.8%	0.085
Body mass index (kg/m^2^), mean ± SD	25.2 ± 4.3	26.9 ± 4.4	24.4 ± 3.9	2.5	1.6 – 3.4	<0.001*
Arterial hypertension, *n* (%)	207 (55.1%)	91 (75.2%)	116 (45.5%)	29.7%	19.9 – 39.5%	<0.001*
Dyslipidemia, *n* (%)	242 (64.4%)	102 (84.3%)	140 (54.9%)	29.4%	20.5 – 38.3%	<0.001*
COPD, *n* (%)	94 (25.0%)	37 (30.6%)	57 (22.4%)	8.2%	–1.5 – 17.9%	0.085
Severe chronic kidney disease^, *n* (%)	40 (10.6%)	17 (14.0%)	23 (9.0%)	5.0%	–2.1 – 12.1%	0.139
**Principal diagnosis for HTX**						
Ischemic CMP, *n* (%)	126 (33.5%)	60 (49.6%)	66 (25.9%)	23.7%	13.3 – 34.1%	<0.001*
Non-ischemic CMP, *n* (%)	187 (49.7%)	53 (43.8%)	134 (52.5%)	8.7%	–2.1 – 19.5%	0.113
Valvular heart disease, *n* (%)	16 (4.3%)	5 (4.1%)	11 (4.3%)	0.2%	–4.1 – 4.5%	0.935
Cardiac amyloidosis, *n* (%)	47 (12.5%)	3 (2.5%)	44 (17.3%)	14.8%	9.4 – 20.2%	<0.001*
**Previous open-heart surgery**						
CABG surgery, *n* (%)	47 (12.5%)	24 (19.8%)	23 (9.0%)	10.8%	2.9 – 18.7%	0.003 *
Other surgery°, *n* (%)	41 (10.9%)	17 (14.0%)	24 (9.4%)	4.6%	–2.5 – 11.7%	0.178
VAD surgery, *n* (%)	29 (7.7%)	11 (9.1%)	18 (7.1%)	2.0%	–4.0 – 8.0%	0.490
**Donor data**						
Age (years), mean ± SD	44.0 ± 12.8	45.0 ± 12.5	43.6 ± 12.9	1.4	–1.4 – 4.2	0.321
Male sex, *n* (%)	126 (33.5%)	43 (35.5%)	83 (32.5%)	3.0%	–7.3 – 13.3%	0.566
Body mass index (kg/m^2^), mean ± SD	25.0 ± 4.5	25.5 ± 3.9	24.8 ± 4.7	0.7	–0.2 – 1.6	0.127
**Transplant sex mismatch**						
Mismatch, *n* (%)	186 (49.5%)	68 (56.2%)	118 (46.3%)	9.9%	–0.9 – 20.7%	0.072
Donor (m) to recipient (f), *n* (%)	12 (3.2%)	6 (5.0%)	6 (2.4%)	2.6%	–1.7 – 6.9%	0.179
Donor (f) to recipient (m), *n* (%)	174 (46.3%)	62 (51.2%)	112 (43.9%)	7.3%	–3.5 – 18.1%	0.184
**Perioperative data**						
Ischemic time (min), mean ± SD	248.1 ± 59.1	250.6 ± 60.7	247.0 ± 58.4	3.6	–9.4 – 16.6	0.588
Biatrial HTX, *n* (%)	5 (1.3%)	1 (0.8%)	4 (1.6%)	0.8%	–1.4 – 3.0%	0.557
Bicaval HTX, *n* (%)	146 (38.8%)	45 (37.2%)	101 (39.6%)	2.4%	–8.1 – 12.9%	0.653
Total orthotopic HTX, *n* (%)	225 (59.9%)	75 (62.0%)	150 (58.8%)	3.2%	–7.3 – 13.7%	0.559

CABG, coronary artery bypass graft; CI, confidence interval; CMP, cardiomyopathy; COPD, chronic obstructive pulmonary disease; f, female; HTX, heart transplantation; m, male; n, number; SD, standard deviation; T2DM, type 2 diabetes mellitus; VAD, ventricular assist device; ^, estimated glomerular filtration rate < 30 ml/min/1.73 m^2^; °, congenital, valvular or ventricular surgery; *, statistically significant (P < 0.050).

Analysis of baseline characteristics stratified by HbA1c at the time of HTX indicated no statistically significant differences between T2DM patients with a HbA1c < 7.0% and T2DM patients with a HbA1c ≥ 7.0% regarding recipient data, principal diagnosis for HTX, previous open-heart surgery, donor data, and perioperative data, except for donor (m) to recipient (f) sex mismatch which was significantly higher in T2DM patients with a HbA1c < 7.0% (*P* = 0.024). Baseline characteristics stratified by HbA1c at HTX are presented in Table 2.

**TABLE 2:** Baseline characteristics – stratified by HbA1c at HTX.

Parameter	T2DM (*n* = 121)	HbA1c < 7.0% (*n* = 67)	HbA1c ≥ 7.0% (*n* = 54)	Difference	95% CI	*P*-value
**Recipient data**						
Age (years), mean ± SD	56.0 ± 7.3	57.0 ± 7.1	54.8 ± 7.5	2.2	–0.4 – 4.8	0.093
Male sex, *n* (%)	99 (81.8%)	51 (76.1%)	48 (88.9%)	12.8%	–0.4 – 26.0%	0.070
Body mass index (kg/m^2^), mean ± SD	26.9 ± 4.4	26.2 ± 4.3	27.6 ± 4.5	1.4	–0.2 – 3.0	0.086
Arterial hypertension, *n* (%)	91 (75.2%)	51 (76.1%)	40 (74.1%)	2.0%	–13.5 – 17.5%	0.796
Dyslipidemia, *n* (%)	102 (84.3%)	57 (85.1%)	45 (83.3%)	1.8%	–11.3 – 14.9%	0.794
COPD, *n* (%)	37 (30.6%)	17 (25.4%)	20 (37.0%)	11.6%	–4.9 – 28.1%	0.166
Severe chronic kidney disease^, *n* (%)	17 (14.0%)	9 (13.4%)	8 (14.8%)	1.4%	–11.1 – 13.9%	0.828
**Principal diagnosis for HTX**						
Ischemic CMP, *n* (%)	60 (49.6%)	35 (52.2%)	25 (46.3%)	5.9%	11.9 – 23.7%	0.516
Non-ischemic CMP, *n* (%)	53 (43.8%)	27 (40.3%)	26 (48.1%)	7.8%	–9.9 – 25.5%	0.387
Valvular heart disease, *n* (%)	5 (4.1%)	3 (4.5%)	2 (3.7%)	0.8%	–6.3 – 7.9%	0.832
Cardiac amyloidosis, *n* (%)	3 (2.5%)	2 (3.0%)	1 (1.9%)	1.1%	–4.3 – 6.5%	0.690
**Previous open-heart surgery**						
CABG surgery, *n* (%)	24 (19.8%)	15 (22.4%)	9 (16.7%)	5.7%	–8.4 – 19.8%	0.433
Other surgery°, *n* (%)	17 (14.0%)	9 (13.4%)	8 (14.8%)	1.4%	–11.1 – 13.9%	0.828
VAD surgery, *n* (%)	11 (9.1%)	6 (9.0%)	5 (9.3%)	0.3%	–10.0 – 10.6%	0.954
**Donor data**						
Age (years), mean ± SD	45.0 ± 12.5	46.1 ± 10.9	43.6 ± 14.3	2.5	–2.2 – 7.2	0.295
Male sex, *n* (%)	43 (35.5%)	25 (37.3%)	18 (33.3%)	4.0%	–13.1 – 21.1%	0.649
Body mass index (kg/m^2^), mean ± SD	25.5 ± 3.9	25.6 ± 4.0	25.3 ± 3.7	0.3	–1.1 – 1.7	0.589
**Transplant sex mismatch**						
Mismatch, *n* (%)	68 (56.2%)	38 (56.7%)	30 (55.6%)	1.1%	–16.7 – 18.9%	0.898
Donor (m) to recipient (f), *n* (%)	6 (5.0%)	6 (9.0%)	0 (0.0%)	9.0%	2.1 – 15.9%	0.024*
Donor (f) to recipient (m), *n* (%)	62 (51.2%)	32 (47.7%)	30 (55.6%)	7.9%	–10.0 – 25.8%	0.394
**Perioperative data**						
Ischemic time (min), mean ± SD	250.6 ± 60.7	245.9 ± 64.3	256.4 ± 56.1	10.5	–11.2 – 32.2	0.338
Biatrial HTX, *n* (%)	1 (0.8%)	0 (0.0%)	1 (1.9%)	1.9%	–1.7 – 5.5%	0.263
Bicaval HTX, *n* (%)	45 (37.2%)	21 (31.3%)	24 (44.4%)	13.1%	–4.2 – 30.4%	0.138
Total orthotopic HTX, *n* (%)	75 (62.0%)	46 (68.7%)	29 (53.7%)	15.0%	–2.3 – 32.3%	0.092

CABG, coronary artery bypass graft; CI, confidence interval; CMP, cardiomyopathy; COPD, chronic obstructive pulmonary disease; f, female; HbA1c, hemoglobin A1c; HTX, heart transplantation; m, male; n, number; SD, standard deviation; T2DM, type 2 diabetes mellitus; VAD, ventricular assist device; ^, estimated glomerular filtration rate < 30 ml/min/1.73 m^2^; °, congenital, valvular or ventricular surgery; *, statistically significant (P < 0.050).

### Medical Treatment of Patients With Type 2 Diabetes Mellitus at Heart Transplantation

Analysis of the immunosuppressive drug therapy showed no statistically significant differences between patients with or without T2DM at the time of HTX regarding the administration of cyclosporine A, tacrolimus, azathioprine, or mycophenolate mofetil (all *P* ≥ 0.050). We also found no statistically significant differences between both groups concerning the administration of acetylsalicylic acid, beta blockers, ivabradine, calcium channel blockers, angiotensin-converting-enzyme inhibitors/angiotensin II receptor blockers, or statins (all *P* ≥ 0.050). Medications after HTX stratified by T2DM at HTX are given in Table 3.

**TABLE 3:** Medications after HTX – stratified by T2DM at HTX.

Parameter	All (*n* = 376)	T2DM (*n* = 121)	No T2DM (*n* = 255)	Difference	95% CI	*P*-value
**Immunosuppressive drug therapy**						
Cyclosporine A, *n* (%)	124 (33.0%)	36 (29.8%)	88 (34.5%)	4.7%	–5.3 – 14.7%	0.359
Tacrolimus, *n* (%)	252 (67.0%)	85 (70.2%)	167 (65.5%)	4.7%	–5.3 – 14.7%	0.359
Azathioprine, n (%)	46 (12.2%)	16 (13.2%)	30 (11.8%)	1.4%	–5.8 – 8.6%	0.687
Mycophenolate mofetil, *n* (%)	330 (87.8%)	105 (86.8%)	225 (88.2%)	1.4%	–5.8 – 8.6%	0.687
Steroids, *n* (%)	376 (100.0%)	121 (100.0%)	255 (100.0%)	0.0%	n. a.	n. a.
**Concomitant medications**						
ASA, *n* (%)	47 (12.5%)	14 (11.6%)	33 (12.9%)	1.3%	–5.8 – 8.4%	0.707
Beta blocker, *n* (%)	85 (22.6%)	25 (20.7%)	60 (23.5%)	2.8%	–6.1 – 11.7%	0.534
Ivabradine, *n* (%)	44 (11.7%)	14 (11.6%)	30 (11.8%)	0.2%	–6.7 – 7.1%	0.956
Calcium channel blocker, *n* (%)	110 (29.3%)	42 (34.7%)	68 (26.7%)	8.0%	–2.1 – 18.1%	0.109
ACE inhibitor/ARB, *n* (%)	159 (42.3%)	52 (43.0%)	107 (42.0%)	1.0%	–9.7 – 11.7%	0.852
Diuretic, *n* (%)	376 (100.0%)	121 (100.0%)	255 (100.0%)	0.0%	n. a.	n. a.
Statin, *n* (%)	211 (56.1%)	73 (60.3%)	138 (54.1%)	6.2%	–4.5 – 16.9%	0.257
Gastric protection ^†^, *n* (%)	376 (100.0%)	121 (100.0%)	255 (100.0%)	0.0%	n. a.	n. a.

*ACE inhibitor, angiotensin-converting-enzyme inhibitor; ARB, angiotensin II receptor blocker; ASA, acetylsalicylic acid; CI, confidence interval; HTX, heart transplantation; n, number; n. a., not applicable; T2DM, type 2 diabetes mellitus; ^†^, gastric protection defined as proton pump inhibitor (PPI) or histamine receptor (H_2_) blocker.*

Likewise, we did not find any statistically significant differences between T2DM patients with a HbA1c < 7.0% and T2DM patients with a HbA1c ≥ 7.0% concerning the immunosuppressive drug therapy or the concomitant medications (all *P* ≥ 0.050). Medications after HTX stratified by HbA1c at HTX are shown in Table 4.

**TABLE 4:** Medications after HTX – stratified by HbA1c at HTX.

Parameter	T2DM (*n* = 121)	HbA1c < 7.0% (*n* = 67)	HbA1c ≥ 7.0% (*n* = 54)	Difference	95% CI	*P*-value
**Immunosuppressive drug therapy**						
Cyclosporine A, *n* (%)	36 (29.8%)	17 (25.4%)	19 (35.2%)	9.8%	–6.6 – 26.2%	0.241
Tacrolimus, *n* (%)	85 (70.2%)	50 (74.6%)	35 (64.8%)	9.8%	–6.6 – 26.2%	0.241
Azathioprine, *n* (%)	16 (13.2%)	9 (13.4%)	7 (13.0%)	0.4%	–11.7 – 12.5%	0.940
Mycophenolate mofetil, *n* (%)	105 (86.8%)	58 (86.6%)	47 (87.0%)	0.4%	–11.7 – 12.5%	0.940
Steroids, *n* (%)	121 (100.0%)	67 (100.0%)	54 (100.0%)	0.0%	n. a.	n. a.
**Concomitant medications**						
ASA, *n* (%)	14 (11.6%)	9 (13.4%)	5 (9.3%)	4.1%	–7.1 – 15.3%	0.476
Beta blocker, *n* (%)	25 (20.7%)	18 (26.9%)	7 (13.0%)	13.9%	–0.1 – 27.9%	0.060
Ivabradine, *n* (%)	14 (11.6%)	9 (13.4%)	5 (9.3%)	4.1%	–7.1 – 15.3%	0.476
Calcium channel blocker, *n* (%)	42 (34.7%)	21 (31.3%)	21 (38.9%)	7.6%	–9.5 – 24.7%	0.386
ACE inhibitor/ARB, *n* (%)	52 (43.0%)	27 (40.3%)	25 (46.3%)	6.0%	–11.7 – 23.7%	0.508
Diuretic, *n* (%)	121 (100.0%)	67 (100.0%)	54 (100.0%)	0.0%	n. a.	n. a.
Statin, *n* (%)	73 (60.3%)	44 (65.7%)	29 (53.7%)	12.0%	–5.5 – 29.5%	0.181
Gastric protection ^†^, *n* (%)	121 (100.0%)	67 (100.0%)	54 (100.0%)	0.0%	n. a.	n. a.

*ACE inhibitor, angiotensin-converting-enzyme inhibitor; ARB, angiotensin II receptor blocker; ASA, acetylsalicylic acid; CI, confidence interval; HbA1c, hemoglobin A1c; HTX, heart transplantation; n, number; n. a., not applicable; T2DM, type 2 diabetes mellitus; ^†^, gastric protection defined as proton pump inhibitor (PPI) or histamine receptor (H_2_) blocker.*

In terms of diabetes medications, metformin was the most common oral anti-diabetic drug in patients with T2DM at the time of HTX (49 of 121 [40.5%]). In addition, almost half of patients with T2DM at the time of HTX received insulin therapy (58 of 121 [47.9%]). Analysis of diabetes medications stratified by HbA1c at the time of HTX showed a significantly higher percentage of regular insulin (*P* = 0.009) and insulin glargine (*P* = 0.028) in T2DM patients with a HbA1c ≥ 7.0%. An overview of the diabetes medications of T2DM patients stratified by HbA1c at HTX is displayed in Table 5.

**TABLE 5:** Overview of diabetes medications.

Parameter	T2DM (*n* = 121)	HbA1c < 7.0% (*n* = 67)	HbA1c ≥ 7.0% (*n* = 54)	Difference	95% CI	*P*-value
**Oral anti-diabetic medications**						
**Alpha glucosidase inhibitors**						
Acarbose, *n* (%)	3 (2.5%)	2 (3.0%)	1 (1.9%)	1.1%	–4.3 – 6.5%	0.690
**Biguanides**						
Metformin, *n* (%)	49 (40.5%)	27 (40.3%)	22 (40.7%)	0.4%	–17.2 – 18.0%	0.961
**DPP-4 inhibitors**						
Saxagliptin, *n* (%)	0 (0.0%)	0 (0.0%)	0 (0.0%)	0.0%	n. a.	n. a.
Sitagliptin, *n* (%)	11 (9.1%)	9 (13.4%)	2 (3.7%)	9.7%	–0.1 – 19.5%	0.064
Vildagliptin, *n* (%)	3 (2.5%)	1 (1.5%)	2 (3.7%)	2.2%	–3.6 – 8.0%	0.437
**GLP-1 receptor agonists**						
Dulaglutide, *n* (%)	0 (0.0%)	0 (0.0%)	0 (0.0%)	0.0%	n. a.	n. a.
Exenatide, *n* (%)	1 (0.8%)	0 (0.0%)	1 (1.9%)	1.9%	–1.7 – 5.5%	0.263
Liraglutide, *n* (%)	2 (1.7%)	1 (1.5%)	1 (1.9%)	0.4%	–4.2 – 5.0%	0.878
**Meglitinides**						
Nateglinide, *n* (%)	0 (0.0%)	0 (0.0%)	0 (0.0%)	0.0%	n. a.	n. a.
Repaglinide, *n* (%)	0 (0.0%)	0 (0.0%)	0 (0.0%)	0.0%	n. a.	n. a.
**SGLT-2 inhibitors**						
Dapagliflozin, *n* (%)	1 (0.8%)	0 (0.0%)	1 (1.9%)	1.9%	–1.7 – 5.5%	0.263
Empagliflozin, *n* (%)	1 (0.8%)	1 (1.5%)	0 (0.0%)	1.5%	–1.4 – 4.4%	0.367
**Sulfonylureas**						
Glibenclamide, *n* (%)	2 (1.7%)	1 (1.5%)	1 (1.9%)	0.4%	–4.2 – 5.0%	0.878
Glimepiride, *n* (%)	12 (9.9%)	8 (11.9%)	4 (7.4%)	4.5%	–6.0 – 15.0%	0.407
Gliquidone, *n* (%)	5 (4.1%)	3 (4.5%)	2 (3.7%)	0.8%	–6.3 – 7.9%	0.832
**Thiazolidinediones**						
Pioglitazone, *n* (%)	0 (0.0%)	0 (0.0%)	0 (0.0%)	0.0%	n. a.	n. a.
Rosiglitazone, *n* (%)	0 (0.0%)	0 (0.0%)	0 (0.0%)	0.0%	n. a.	n. a.
**Insulin therapy**						
**Rapid-acting insulin**						
Insulin aspart, *n* (%)	9 (7.4%)	5 (7.5%)	4 (7.4%)	0.1%	–9.3 – 9.5%	0.991
Insulin glulisine, *n* (%)	1 (0.8%)	0 (0.0%)	1 (1.9%)	1.9%	–1.7 – 5.5%	0.263
Insulin lispro, *n* (%)	3 (2.5%)	1 (1.5%)	2 (3.7%)	2.2%	–3.6 – 8.0%	0.437
**Short-acting insulin**						
Regular insulin, *n* (%)	45 (37.2%)	18 (26.9%)	27 (50.0%)	23.1%	6.1 – 40.1%	0.009*
**Intermediate-acting insulin**						
NPH insulin, *n* (%)	13 (10.7%)	6 (9.0%)	7 (13.0%)	4.0%	–7.3 – 15.3%	0.479
**Long-acting insulin**						
Insulin degludec, *n* (%)	0 (0.0%)	0 (0.0%)	0 (0.0%)	0.0%	n. a.	n. a.
Insulin detemir, *n* (%)	4 (3.3%)	1 (1.5%)	3 (5.6%)	4.1%	–2.7 – 10.9%	0.214
Insulin glargine, *n* (%)	41 (33.9%)	17 (25.4%)	24 (44.4%)	19.0%	2.2 – 35.8%	0.028*

CI, confidence interval; DPP-4, dipeptidyl peptidase-4; GLP-1, glucagon-like peptide-1; HbA1c, hemoglobin A1c; n, number; n. a., not applicable; NPH, Neutral Protamine Hagedorn; SGLT-2, sodium-glucose transport protein 2; T2DM, type 2 diabetes mellitus; *, statistically significant (P < 0.050).

### Survival of Patients With Type 2 Diabetes Mellitus at Heart Transplantation

Patients with T2DM at the time of HTX had a significantly higher 5-year all-cause mortality after HTX (41.3% versus 29.8%, difference: 11.5%, 95% CI: 1.1 – 21.9%, *P* = 0.027). Regarding the causes of death within 5 years after HTX, significantly more patients with T2DM died from graft failure (14.9% versus 7.8%, difference: 7.1%, 95% CI: 0.1 – 14.1%, *P* = 0.035). For further evaluation of the association between T2DM and graft failure, we performed a log rank test between patients with and without T2DM at HTX in regard to graft failure within 5 years after HTX analyzing the number of patients with graft failure and the time from HTX until graft failure. Patients with T2DM at the time of HTX had a significantly higher rate of graft failure within 5 years after HTX (*P* = 0.019). In contrast, we did not observe statistically significant differences between T2DM groups concerning acute rejection, infection/sepsis, malignancy, or thromboembolic event/bleeding (all *P* ≥ 0.050). Causes of death within 5 years after HTX stratified by T2DM at HTX are given in Table 6.

**TABLE 6:** Causes of death within 5 years after HTX – stratified by T2DM at HTX.

Parameter	All (*n* = 376)	T2DM (*n* = 121)	No T2DM (*n* = 255)	Difference	95% CI	*P*-value
Graft failure, *n* (%)	38 (10.1%)	18 (14.9%)	20 (7.8%)	7.1%	0.1 – 14.1%	0.035*
Acute rejection, *n* (%)	4 (1.1%)	1 (0.8%)	3 (1.2%)	0.4%	–1.7 – 2.5%	0.757
Infection/Sepsis, *n* (%)	66 (17.5%)	20 (16.5%)	46 (18.0%)	1.5%	–6.6 – 9.6%	0.719
Malignancy, *n* (%)	8 (2.1%)	5 (4.1%)	3 (1.2%)	2.9%	–0.9 – 6.7%	0.064
Thromboembolic event/bleeding, *n* (%)	10 (2.7%)	6 (5.0%)	4 (1.6%)	3.4%	–0.8 – 7.6%	0.056
All causes, *n* (%)	126 (33.5%)	50 (41.3%)	76 (29.8%)	11.5%	1.1 – 21.9%	0.027*

CI, confidence interval; HTX, heart transplantation; n, number; T2DM, type 2 diabetes mellitus; *, statistically significant (P < 0.050).

Analysis of causes of death within 5 years after HTX stratified by HbA1c at the time of HTX showed a significantly higher 5-year all-cause mortality after HTX in T2DM patients with a HbA1c ≥ 7.0% (53.7% versus 31.3%, difference: 22.4%, 95% CI: 5.1–39.7%, *P* = 0.013). Patients with T2DM and a HbA1c ≥ 7.0% at the time of HTX also had a higher percentage of death due to graft failure (18.5% versus 11.9%), infection/sepsis (24.1% versus 10.5%), and thromboembolic event/bleeding (9.3% versus 1.5%) within 5 years after HTX. Causes of death within 5 years after HTX stratified by HbA1c at HTX are provided in Table 7.

**TABLE 7:** Causes of death within 5 years after HTX – stratified by HbA1c at HTX.

Parameter	T2DM (*n* = 121)	HbA1c < 7.0% (*n* = 67)	HbA1c ≥ 7.0% (*n* = 54)	Difference	95% CI	*P*-value
Graft failure, *n* (%)	18 (14.9%)	8 (11.9%)	10 (18.5%)	6.6%	–6.3 – 19.5%	0.312
Acute rejection, *n* (%)	1 (0.8%)	1 (1.5%)	0 (0.0%)	1.5%	–1.4 – 4.4%	0.367
Infection/Sepsis, *n* (%)	20 (16.5%)	7 (10.5%)	13 (24.1%)	13.6%	0.1 – 27.1%	0.045*
Malignancy, *n* (%)	5 (4.1%)	4 (6.0%)	1 (1.9%)	4.1%	–2.6 – 10.8%	0.258
Thromboembolic event/bleeding, *n* (%)	6 (5.0%)	1 (1.5%)	5 (9.3%)	7.8%	–0.4 – 16.0%	0.050
All causes, *n* (%)	50 (41.3%)	21 (31.3%)	29 (53.7%)	22.4%	5.1 – 39.7%	0.013*

CI, confidence interval; HbA1c, hemoglobin A1c; HTX, heart transplantation; n, number; T2DM, type 2 diabetes mellitus; *, statistically significant (P < 0.050).

Multivariate analysis showed a more than 50% increased risk of death within 5 years after HTX in patients with T2DM at the time of HTX (HR: 1.563; 95% CI: 1.053–2.319; *P* = 0.027), while the other seven included variables (recipient age, recipient body mass index, recipient arterial hypertension, recipient dyslipidemia, previous CABG surgery, ischemic CMP as principal diagnosis for HTX, and cardiac amyloidosis as principal diagnosis for HTX) showed no statistically significant effect on 5-year post-transplant mortality. Multivariate analysis for 5-year mortality after HTX is given in Table 8.

**TABLE 8:** Multivariate analysis for 5-year mortality after HTX.

Parameter	Hazard Ratio	95% CI	*P*-value
Recipient age (years)	1.018	0.996 – 1.041	0.103
Recipient body mass index (kg/m^2^)	1.012	0.969 – 1.058	0.592
Recipient arterial hypertension (in total)	0.704	0.404 – 1.228	0.217
Recipient dyslipidemia (in total)	0.935	0.548 – 1.596	0.805
Recipient T2DM (in total)	1.563	1.053 – 2.319	0.027 *
Previous CABG surgery (in total)	0.783	0.434 – 1.412	0.415
Ischemic CMP (in total)	1.718	0.993 – 2.972	0.053
Cardiac amyloidosis (in total)	1.697	0.985 – 2.923	0.057

CABG, coronary artery bypass graft; CI, confidence interval; CMP, cardiomyopathy; HTX, heart transplantation; T2DM, type 2 diabetes mellitus; *, statistically significant (P < 0.050).

**FIGURE 1:**
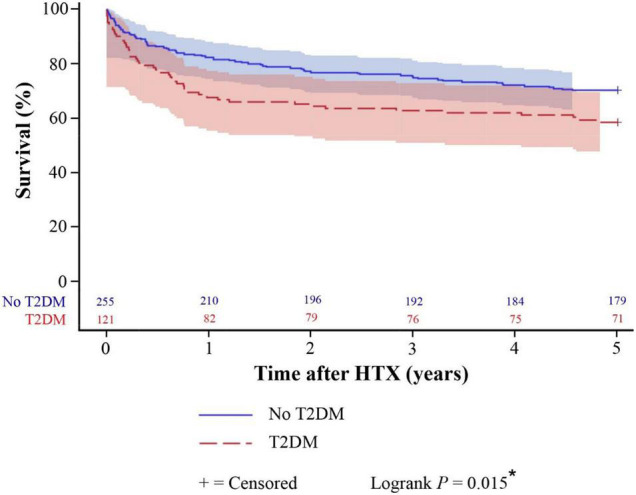
Five-year post-transplant survival of patients with and without T2DM at HTX (Kaplan–Meier estimator). Patients with T2DM at HTX had a significantly worse 5-year post-transplant survival in the Kaplan–Meier survival analysis (58.7%) compared to patients without T2DM at HTX (70.2%, *P* = 0.015). HTX, heart transplantation; T2DM, type 2 diabetes mellitus; *, statistically significant (*P* < 0.050).

**FIGURE 2:**
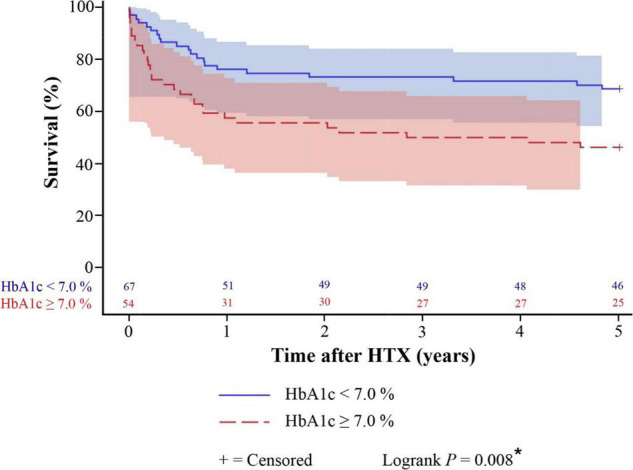
Five-year post-transplant survival of patients with T2DM stratified by HbA1c at HTX (Kaplan–Meier estimator). Stratification of patients with T2DM at HTX showed a significantly lower 5-year post-transplant survival of patients with a HbA1c ≥ 7.0% at HTX (46.3%) in comparison to patients with a HbA1c < 7.0% at HTX (68.7%, *P* = 0.008). HbA1c, hemoglobin A1c; HTX, heart transplantation; T2DM, type 2 diabetes mellitus; *, statistically significant (*P* < 0.050).

Kaplan–Meier survival analysis displayed a significantly inferior 5-year post-transplant survival of patients with T2DM at the time of HTX (58.7%) in comparison to patients without T2DM at the time of HTX (70.2%, difference: 11.5%, 95% CI: 1.1 – 21.9%, *P* = 0.015). Patients with insulin therapy had in fact a lower 5-year post-transplant survival (53.4%) than patients without insulin therapy (63.5%) but this difference did not reach statistical significance (*P* = 0.243). Further stratification of T2DM patients at the time of HTX showed a significantly lower 5-year post-transplant survival of patients with a HbA1c ≥ 7.0% at HTX (46.3%) in comparison to patients with a HbA1c < 7.0% at the time of HTX (68.7%, difference: 22.4%, 95% CI: 5.1–39.7%, *P* = 0.008). Kaplan–Meier estimators are shown in Figures 1, 2.

**FIGURE 3:**
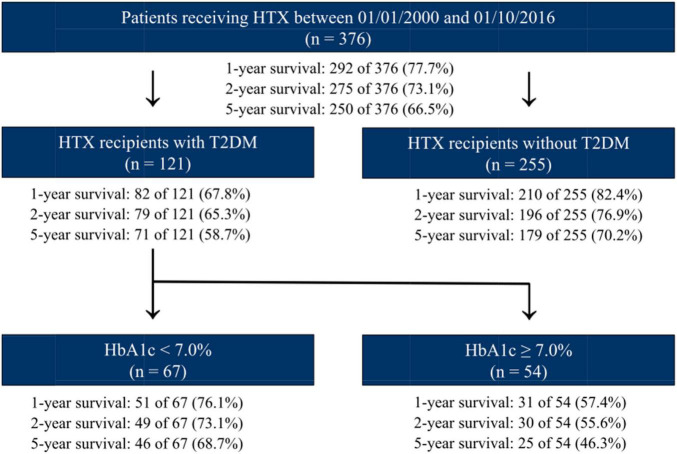
Overview of 5-year post-transplant survival stratified by T2DM at HTX and HbA1c at HTX. Patients without T2DM at HTX showed the best 1-, 2-, and 5-year post-transplant survival, followed by patients with T2DM at HTX and a HbA1c < 7.0% at HTX, whereas patients with T2DM at HTX and a HbA1c ≥ 7.0% at HTX had the worst 1-, 2-, and 5-year post-transplant survival. HbA1c, hemoglobin A1c; HTX, heart transplantation; n, number; T2DM, type 2 diabetes mellitus.

Additional survival analysis revealed that patients without T2DM at the time of HTX had the best 1-year (210 of 255 [82.4%]), 2-year (196 of 255 [76.9%]), and 5-year post-transplant survival (179 of 255 [70.2%]), followed by patients with T2DM at HTX and a HbA1c < 7.0% at HTX who showed a broadly similar 1-year (51 of 67 [76.1%]), 2-year (49 of 67 [73.1%]), and 5-year post-transplant survival (46 of 67 [68.7%]). Of note, patients with T2DM at HTX and a HbA1c ≥ 7.0% at HTX had the worst 1-year (31 of 54 [57.4%]), 2-year (30 of 54 [55.6%]), and 5-year post-transplant survival (25 of 54 [46.3%]). An overview of 5-year post-transplant survival stratified by T2DM at HTX and HbA1c at HTX is provided in Figure 3.

### Sensitivity Analysis

In order to investigate a possible era effect and to examine the robustness of our findings, we performed a sensitivity analysis with a subgroup of patients who were administered tacrolimus and mycophenolate mofetil as immunosuppressive drug therapy [252 of 376 patients (67.0%)]. This analysis provided similar results supporting the robustness of our findings and reducing the likelihood of a potential era effect.

## Discussion

### Frequency and Clinical Relevance of Type 2 Diabetes Mellitus at Heart Transplantation

Given the unknown prognostic effect of T2DM at the time of HTX on post-transplant outcomes, we performed this large study with 376 HTX recipients to investigate the frequency and clinical relevance of pre-transplant T2DM. A total of 121 HTX recipients (32.2%) had pre-transplant T2DM. This is in line with recent studies describing a similar percentage of pre-transplant diabetic patients (24, 25). Chamarthi et al. (24) reported a pre-transplant diabetic rate of 28.8% (46 of 160) at Brigham and Women’s Hospital, Harvard Medical School, between January 2000 and July 2012. Similarly, Feng et al. (25) published a pre-transplant diabetic rate of 30.7% (75 of 244) at Stanford University Medical Center between January 2008 and July 2018. In contrast, older studies covering earlier eras of HTX reported a considerably lower rate of pre-transplant diabetic patients ranging between 13.7 and 18.3% (22, 23). These data evidently highlight the rising percentage of diabetic HTX recipients (22–25).

From a clinician’s perspective, this change is of high relevance as patients with T2DM face an increased risk of morbidity and mortality (1–8, 13–16, 36). Diabetic patients have a higher risk for post-transplant infections requiring hospitalization and often suffer from further deterioration of renal function, especially in combination with calcineurin inhibitors which are known nephrotoxic drugs (20, 21, 25, 37–39). In order to evaluate the degree of end-organ damage as well as the clinical status of patients at the time of HTX, we compared patients with and without T2DM at the time of HTX. We found no statistically significant differences between both groups in regard to severe chronic kidney disease, total bilirubin, hemoglobin, hospitalization before HTX, days on waiting list, high urgency status on waiting list, inotropic support, intra-aortic balloon pump, or initial hospital stay after HTX highlighting the importance of careful evaluation of T2DM patients before listing for HTX. Alternatively, patients with T2DM and severe end-organ damage may be considered for left ventricular assist device (LVAD) implantation (21). However, especially in younger patients, this may not be a valid long-term solution given the known LVAD complications (40, 41). Hence, patients with T2DM should be carefully evaluated before listing for HTX, particularly in regard to severe end-organ damage, and should receive optimal individualized diabetes management before and after HTX (1–8).

### Clinical Management of Patients With Type 2 Diabetes Mellitus at Heart Transplantation

In order to reduce microvascular and macrovascular complications in patients with T2DM, the 2019 European Society of Cardiology (ESC) guidelines on diabetes recommend a targeted HbA1c < 7.0% (1). We therefore compared in our study the diabetes medications of T2DM patients with a HbA1c < 7.0% and T2DM patients with a HbA1c ≥ 7.0%. We found no significant difference between both groups in regard to oral anti-diabetic medications of which metformin was the most common oral anti-diabetic drug in patients with T2DM at the time of HTX. In terms of insulin therapy which was administered to almost half of patients with T2DM at the time of HTX, T2DM patients with a HbA1c ≥ 7.0% had a significantly higher percentage of regular insulin (*P* = 0.009) and insulin glargine (*P* = 0.028).

With the introduction of new anti-diabetic medications such as dipeptidyl peptidase-4 (DPP-4) inhibitors, glucagon-like peptide-1 (GLP-1) receptor agonists, and sodium-glucose transport protein 2 (SGLT-2) inhibitors, diabetes management has improved and a targeted HbA1c < 7.0% has become more achievable (1, 3, 25, 42–48). In addition to their excellent glucose-lowering profile, these novel agents exhibit multiple beneficial effects *via* reduction of body weight, blood pressure, major cardiovascular events and even mortality (1, 3, 25, 42–48). However, data regarding the safety and efficacy of these new drugs in HTX recipients with T2DM are limited to studies with small sample sizes (42, 45).

Cehic et al. (42) examined 22 HTX recipients with T2DM who were treated with empagliflozin. They observed no genitourinary infections and treatment with empagliflozin was associated with reductions in body mass index and HbA1c (42). Similar findings were reported by Sammour et al. (45) who evaluated the safety and efficacy of GLP-1 receptor agonists and SGLT-2 inhibitors in HTX recipients with T2DM. Among 21 patients, they found a significant reduction of body weight, HbA1c, and low-density lipoprotein-cholesterol with no adverse events leading to discontinuation of either therapy (45).

In our study, only a minority of patients with pre-transplant T2DM received DPP-4 inhibitors, GLP-1 receptor agonists, or SGLT-2 inhibitors, as the majority of patients with oral anti-diabetic medications were still on metformin. This is in line with a recent study by Feng et al. (25) reporting likewise only a few HTX recipients on GLP-1 receptor agonists or SGLT-2 inhibitors. Further studies with large contemporary populations of HTX recipients with T2DM are therefore needed to determine the safety and efficacy of these medications but one should keep in mind that the use of anti-diabetic medications is just one part of diabetes management. A multimodal approach including nutrition counseling, increased physical activity, weight loss, smoking cessation in addition to anti-diabetic medications with new pharmacologic strategies is required to reduce the burden of morbidity and mortality in HTX recipients with T2DM.

### Post-transplant Mortality of Patients With Type 2 Diabetes Mellitus at Heart Transplantation

Data regarding the impact of pre-transplant T2DM on mortality after HTX are inconclusive (9–21). Several studies reported an increased post-transplant mortality in patients with T2DM at the time of HTX (13–16), whereas others studies did not find a relevant difference (17–21).

In our study with a large contemporary population of HTX recipients, patients with pre-transplant T2DM had a significantly increased 5-year all-cause mortality after HTX (41.3% versus 29.8%, *P* = 0.027), along with a higher rate of death due to graft failure (14.9% versus 7.8%, *P* = 0.035). Multivariate analysis showed a more than 50% increased risk for 5-year mortality after HTX in these patients (HR: 1.563; 95% CI: 1.053–2.319; *P* = 0.027).

Discrepancies between former studies regarding post-transplant mortality in patients with T2DM at the time of HTX may result from differences in diabetes status (13–21). A large study of the United Network of Organ Sharing (UNOS) database with 20,412 HTX recipients including 3,687 diabetic patients reported a significantly better post-transplant survival in non-diabetic HTX recipients than in diabetic HTX recipients in general (*P* < 0.001) but there was no statistically significant difference in post-transplant survival between non-diabetic HTX recipients and diabetic HTX recipients with uncomplicated diabetes status (*P* = 0.080) (21).

Stratification of patients with T2DM by HbA1c at HTX in our study showed a significantly higher 5-year all-cause mortality after HTX in T2DM patients with a HbA1c ≥ 7.0% (53.7% versus 31.3%, *P* = 0.013). Patients with T2DM and a HbA1c ≥ 7.0% also had a higher percentage of death due to graft failure (18.5% versus 11.9%), infection/sepsis (24.1% versus 10.5%), and thromboembolic event/bleeding (9.3% versus 1.5%) within 5 years after HTX highlighting the vulnerability of these patients. As insulin therapy is often needed in patients with advanced T2DM, we also compared patients with and without insulin therapy. HTX recipients with insulin therapy had in fact a lower 5-year post-transplant survival (53.4%) than patients without insulin therapy (63.5%) but this difference did not reach statistical significance (*P* = 0.243). This is in line with a report by Czerny et al. (13) who also found no significant influence of insulin therapy on survival after HTX.

Furthermore, a key message of our study is the finding that patients with T2DM and a HbA1c < 7.0% had a similar 5-year survival after HTX in comparison to patients without T2DM indicating that comparable long-term post-transplant survival rates of HTX recipients with T2DM are achievable if these patients receive optimal diabetes management and are followed-up closely after HTX.

Regarding the impact of diabetes on ventricular ejection fraction and cardiac allograft vasculopathy, results have been controversially discussed (4, 11, 13, 17, 49). Higgins et al. (17) reported that diabetic HTX recipients had an increased rate of transplant coronary artery disease (42% versus 13%; *P* = 0.02) as well as a lower left ventricular ejection fraction at 3 years after HTX (54% versus 61%; *P* = 0.03). In contrast, Munoz et al. (11) found no statistically significant difference in transplant coronary artery disease by the fourth year of follow-up (31% in diabetic HTX recipients versus 33% in non-diabetic HTX recipients). Similar results were reported by Czerny et al. (13) who also reported no statistically significant difference in transplant coronary artery disease (15% in diabetic HTX recipients versus 14% in non-diabetic HTX recipients) at 5 years after HTX.

In our study, survival of T2DM patients declined markedly within the first year after HTX, some patients with T2DM even died from graft failure within the first 3 months after HTX. As the development of cardiac allograft vasculopathy usually takes several months to years after HTX this may indicate that adverse graft survival in HTX recipients is rather related to generally impaired global organ function (13). However, given the importance of this aspect and the lack of contemporary knowledge, there is an urgent need for future studies focusing on the development of cardiac allograft vasculopathy by analyzing catheterization data of HTX recipients with T2DM.

### Study Limitations

The findings of this study were derived from a large single-center registry (Heidelberg HTX Registry) including the highly elaborated data of 376 patients who received HTX at Heidelberg Heart Center. Given the known limitations of such a study design, our results should be interpreted carefully and within the context of the existing literature. However, we would like to point out that our study was comparable to multicenter studies in sample size and our patients received standardized treatment and follow-up, decreasing the likelihood of potential selection bias and confounders (26–35).

In order to detect long-term effects of T2DM in HTX recipients, we selected adult HTX recipients who received HTX at Heidelberg Heart Center between 01/01/2000 and 01/10/2016, enabling a minimum post-transplant follow-up of 5 years. This study included data of HTX recipients over a period of more than 20 years. A possible era effect as a result of changes in medical and surgical care may have therefore affected our results. As tacrolimus replaced cyclosporine A as the main immunosuppressive agent from 2006 onward, we investigated a possible era effect by comparing the immunosuppressive drug therapy of HTX recipients with or without T2DM. We could neither detect a statistically significant difference between HTX recipients with or without T2DM regarding the use of cyclosporine A or tacrolimus, nor concerning the use of azathioprine or mycophenolate mofetil supporting the robustness of our findings (26–35).

Our results should be interpreted as hypothesis-generating, especially in the context of post-transplant survival. We can therefore neither proof nor disproof a causal relationship between T2DM at the time of HTX and increased 5-year post-transplant mortality but merely indicate an association. In addition, the effects of the recently introduced SGLT-2 inhibitors on long-term post-transplant mortality in HTX recipients remain unknown and require further investigation, preferably in form of large multicenter trials.

## Conclusion

The number of HTX recipients with pre-transplant T2DM has continuously been growing over the last decades. Many of these patients suffer from impaired wound healing, infections, renal dysfunction, thromboembolic complications, cardiac rejections, and cardiac allograft vasculopathy. Management of HTX recipients with T2DM is therefore very challenging but data about this topic are still very limited. In order to investigate the effects of pre-transplant T2DM on survival and causes of death after HTX, we performed a large study with a contemporary population of 376 HTX recipients including 121 patients with T2DM (32.2%). We observed a significantly higher 5-year all-cause mortality after HTX in patients with pre-transplant T2DM (41.3% versus 29.8%, *P* = 0.027) along with a higher percentage of death due to graft failure (14.9% versus 7.8%, *P* = 0.035). Multivariate analysis indicated pre-transplant T2DM as a significant risk factor for 5-year mortality after HTX (HR: 1.563; 95% CI: 1.053–2.319; *P* = 0.027). Stratification of HTX recipients with pre-transplant T2DM showed no statistically significant difference in 5-year survival between patients with and without insulin therapy (*P* = 0.243) but patients with pre-transplant T2DM and a HbA1c < 7.0% had a significantly better 5-year survival than patients with a HbA1c ≥ 7.0% (*P* = 0.008). Of note, patients with T2DM and a HbA1c < 7.0% had a similar 5-year survival after HTX compared to patients without T2DM. Therefore, patients with T2DM can successfully undergo HTX if they receive optimal diabetes management before and after HTX.

## Data Availability Statement

The original contributions presented in this study are included in the article/supplementary material, further inquiries can be directed to the corresponding author/s.

## Ethics Statement

The studies involving human participants were reviewed and approved by Institutional Review Board (IRB) of Heidelberg University, Germany (ethical approval number: S-286/2015, Version 1.2, 28-07-2020). The patients/participants provided their written informed consent to participate in this study.

## Author Contributions

RR, CG, TB, and LK: conceptualization, methodology, and investigation. RR, CG, MH, FD, TB, and LK: formal analysis, validation, and data curation. RR, CG, and LK: writing – original draft preparation, review and editing. RR, CG, FD, and LK: visualization. RR and FD: funding acquisition. RR, PE, WS, GW, SK, JS, NF, and LK: supervision and resources. All authors: contributed to the article and approved the submitted version.

## Conflict of Interest

The authors declare that the research was conducted in the absence of any commercial or financial relationships that could be construed as a potential conflict of interest.

## Publisher’s Note

All claims expressed in this article are solely those of the authors and do not necessarily represent those of their affiliated organizations, or those of the publisher, the editors and the reviewers. Any product that may be evaluated in this article, or claim that may be made by its manufacturer, is not guaranteed or endorsed by the publisher.
